# A Fusion Recognition Method Based on Multifeature Hidden Markov Model for Dynamic Hand Gesture

**DOI:** 10.1155/2020/8871605

**Published:** 2020-09-09

**Authors:** Guoliang Chen, Kaikai Ge

**Affiliations:** School of Mechanical and Electronic Engineering, Wuhan University of Technology, Wuhan 430070, Hubei, China

## Abstract

In this paper, a fusion method based on multiple features and hidden Markov model (HMM) is proposed for recognizing dynamic hand gestures corresponding to an operator's instructions in robot teleoperation. In the first place, a valid dynamic hand gesture from continuously obtained data according to the velocity of the moving hand needs to be separated. Secondly, a feature set is introduced for dynamic hand gesture expression, which includes four sorts of features: palm posture, bending angle, the opening angle of the fingers, and gesture trajectory. Finally, HMM classifiers based on these features are built, and a weighted calculation model fusing the probabilities of four sorts of features is presented. The proposed method is evaluated by recognizing dynamic hand gestures acquired by leap motion (LM), and it reaches recognition rates of about 90.63% for LM-Gesture3D dataset created by the paper and 93.3% for Letter-gesture dataset, respectively.

## 1. Introduction

Dynamic hand gesture recognition is a very intriguing problem in recent years that, if efficiently solved, could be the wealthiest means of communication that can be used. Because of this, many scholars from all over the world have done a lot of theoretical and practical research studies [1]. Compared with static gestures, the meaning of dynamic gestures is more abundant, and it is more common and natural to be an interactive way. But, at the same time, the information of dynamic hand gestures, such as shape and location, varies as time, which consequently increases the difficulty in recognition.

At present, there are two main types of sensors that are capable of sensing hand gestures: wearable sensor or vision-based sensor [2, 3]. The former approach could capture the movement of hands and fingers at the expense of convenience and cost and sufficiently extract information of hand, but it places an additional burden on users and could feel unnatural enough to perform hand gestures. Some advantages of a vision-based sensor are it can be less cumbersome and has more natural interaction than the wearable sensor due to no physical contact with users. However, its computational complexity is quite high for hand detecting, tracking, and extracting [4]. For instance, a hand should be separated from the background before the final recognition, which can be significantly affected by external environmental factors like ambient light. On the contrary, due to the complex 3D movements of hands or fingers, it is difficult to properly understand the performed hand pose based on the extracted information from 2D images [5]. Besides, once the palm surface is not parallel to the camera, for example, the recognition work could be harder.

The classification is a crucial step to recognize hand gestures. Five main classifying methods of hand gesture based on 3D vision can be identified: support vector machines (SVMs), artificial neural network (ANN), template matching (TM), HMM, and dynamic time warping (DTW) [4]. The SVM is a popular classifier for hand gesture recognition, in which support vectors are used to determine the hyperplane to realize the maximum separation of the hand gesture classes [6]. In vision-based hand gesture recognition systems, the ANN is used as a classifier to handle only fundamental and limited hand gestures [7]. When the high-level discriminative 3D hand features are available, the TM is an excellent choice for recognizing hand gestures, which works quite well with the contour- or boundary-based hand features [8]. As the hand gesture is a continuous pattern concerning time, the HMM is found to be the most suitable pattern recognition tool for testing on a moderately large dataset [9]. DTW is an indirect continuous hand gesture recognition approach that automatically aligns the sequences with different lengths and returns the proper distance [10].

Martin Sagayam and Jude Hemanth [11] develop a probabilistic model based on the state sequence analysis in the HMM to recognize hand gestures taken from the Cambridge hand dataset. The experimental results show that the proposed method achieves a 0.98% reduction in error rate and a 1.55% improvement in the recognition rate over that of the Viterbi prediction. Some work combines HMM with other methods for gesture recognition. Zhou et al. [12] use HMM to model the different information sequences of dynamic hand gestures and use BP neural network (BPNN) as a classifier to process the resulting hand gestures modeled by HMM, which achieves a satisfactory real-time performance and an accuracy above 84%. Martin Sagayam and Jude Hemanth [13] propose a hybrid 1D HMM model with artificial bee colony (ABC) optimization. The method is carried out with nine different classes of hand gestures that are used for virtual reality applications. The experimental results show that the average value of the recognition rate with ABC optimization increases by 2.72%, and the average value of the error rate is decreased by 0.47%.

With the emergence and development of deep learning technology, some scholars try to apply the technology for hand gesture recognition. Oyedotun and Khashman [14] apply a convolutional neural network (CNN) and stacked denoising autoencoder (SDAE) to recognize 24 American Sign Language (ASL) hand gestures obtained from a public database, which achieves the recognition rates of 91.33 and 92.83%. Bao et al. [15] propose a deep CNN that can classify hand gestures from the whole image without any segmentation or detection stage information. The method can organize seven sorts of hand gestures in a user-independent manner and achieve an accuracy of 97.1% in the dataset with simple backgrounds and 85.3% in the dataset with complex backgrounds.

In recent years, 3D sensors, such as binocular cameras, Kinect, and LM, have been applied for hand gesture recognition with excellent performance. LM can detect and track hands and fingers with an accuracy of about 0.01 mm and feedback the gesture information in real time with a sampling rate of 120 fps [16]. Because of its superior performance, many researchers consider that it is a promising 3D sensor and particularly suitable for hand gesture recognition. For instance, Chen et al. [17] extract directional codes of 3D motion trajectory as the feature and exploit a classifier based on SVM to classify letter and number gestures. Ameur et al. [18] extract the positions of fingertips and palm center as features that are then trained with an SVM classifier. Their method reaches an average recognition rate of about 81% with 11 kinds of dynamic gestures. Xu et al. [19] and Zeng et al. [20] also conducted similar studies. Besides *t*, some researchers are working on dynamic gesture recognition. Lu et al. [21] build two kinds of features and feed them into the hidden conditional neural field classifier to recognize dynamic gestures. Avola et al. [22] propose a long short-term memory (LSTM) and recurrent neural networks (RNNs) combined with an effective set of discriminative features based on both joint angles and fingertip positions to recognize sign language and semaphoric hand gestures, which achieves an accuracy of over 96%. Vamsikrishna et al. [9] propose a low-cost computer-vision-assisted setup based on LM to detect precise movements of palm or finger within the field of view of the sensors. Then, it presents a set of discrete HMM for classifying the gesture sequences performed during rehabilitation.

The paper is aimed at recognizing the hand gestures corresponding to an operator's hand commands in robot teleoperation. For the problem, the paper develops four feature vectors and their extraction models based on 3D information acquired by LM to describe the hand gestures. And then, the article establishes HMMs to calculate the occurrence probabilities of four feature sequences in an unknown hand gesture, respectively. Lastly, the paper uses a weighted algorithm to fuse the occurrence probabilities of four features. The most considerable hazard is taken as can be taken as a recognition result. The rest of the paper is organized as follows. Prophase works of hand gesture recognition are introduced in Section 2. The methods of feature extraction are presented in Section 3, including valid dynamic gesture judgment, feature definition, and feature sequence clustering. HMM training model and hand gesture recognition by fusing the feature probabilities are proposed in Section 4.  Section 5 comprises experiments and the result and discussion. Conclusion and possible future extensions are given in Section 6.

## 2. Prophase Work of Gesture Recognition

### 2.1. Leap Motion and Data Acquisition

LM, based on time-of-flight technology, mainly consists of three infrared LEDs and two infrared cameras, which can take photos from different directions to obtain gesture information in 3D space [16]. LM has about 150 degrees view field and an effective range of approximately 0.03 to 0.06 meters above itself. LM could feedback data frames that consist of positions and velocities of key points, rotation information, and frame timestamp.

**Figure 1:**
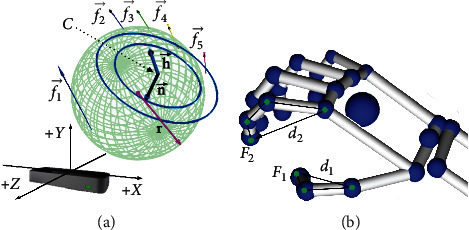
Data acquisition from leap motion.

When collecting gestures, LM will establish a right-hand coordinate system, as shown in Figure 1, based on all obtained data such as position, speed, and gesture of human hands. As shown in Figure 1, the five fingertips are denoted by *f*_*i*_(*i*=1,…, 5), and palm center is denoted by *C*. We mainly focus on the following data: (1) palm normal vector $\vec{n}$ and palm direction vector $\vec{h}$, which represent unit vectors perpendicular to the palm plane and point from the palm position toward the fingers, respectively; (2) finger direction vector ${\vec{f}}_{i}$ and the finger extension length points *d*_*i*_, which represent the unit vector pointing to the point of the finger point *F*_*i*_ and the distance between two points, respectively; (3) instantaneous velocity *v*_*i*_ of five fingertips and instantaneous velocity *v*_*C*_ of the palm center; and (4) coordinate *p*_*t*_(*x*_*t*_, *y*_*t*_, *z*_*t*_), which represents the coordinate of the palm position in the frame *t*.

### 2.2. Dynamic Gesture Definition

There are relatively few publicly available hand gesture datasets created by LM-sampled images, especially for dynamic hand gestures in robot teleoperation. We analyze the movement characteristics of the operator's hand command in the robot teleoperation, such as translation and rotation of three degrees of freedom, and create a gesture dataset named LM-Gesture3D, which contains eight different dynamic gestures, as shown in Table 1. All these gestures collected by LM represent some practical operations or command signs and can be performed easily and naturally. Besides, there are similarities among the gestures in some respects, which will be illuminated in more detail later.

## 3. Feature Extraction

### 3.1. Valid Dynamic Gesture Judgment

Despite the fact that LM has many merits, it mainly acts as a gesture data collector similar to a wearable device and camera. Hence, conditions for judging the beginning and the end of a valid dynamic gesture need to be given first. Take LM-Gesture3D as an example; it can be seen that the fingertips and palm center will inevitably produce rapid and continuous displacement when either gesture is performed. Even for a simplest dynamic gesture, click, for example, is no exception. A simple discriminant, based on the above analysis, is established as follows:

(1) $$\begin{array}{c} v=\max\left\{v_{C},v_{i}|i=1,\ldots ,5\right\}>v_{\tau }, \end{array}$$

where *v*_*C*_ and *v*_*i*_ are the instantaneous velocity of palm center and fingertips, respectively, and *v*_*τ*_ is the predefined velocity threshold.

When the total number of continuous frames up to 60, *v*_*C*_ and *v*_*i*_, satisfy discriminant (1), the data frames will be regarded as the original data of a valid dynamic gesture.

As LM is quite sensitive, in both cases when hand makes a slight shaking at rest and the obtained data contain noise, discriminant (1) could be satisfied in a few consecutive frames. So the total number (i.e., 60 frames) is set to eliminate these useless data. In addition, dynamic gesture with a low speed will be judged as invalid by discriminant (1), which means there is a degree of freedom for hand movement.

### 3.2. Feature Definition

To effectively recognize dynamic gestures, changes in hand posture and position are analyzed separately. The former can be further divided into the bending angle of fingers, opening perspective between fingers, and palm posture. The gesture trajectory can be represented later. Therefore, the paper describes the changes in gestures through the above four features.

The specific extraction process and expression of the four features are as follows.

#### 3.2.1. Palm Attitude Feature

If the palm shape changes little in a dynamic gesture, the change in palm posture can be regarded as the problem of attitude angle calculation of a rigid body. The paper draws lessons from the 3D attitude measurement method, which is pointed out in [23].

As shown in Figure 1, the palm posture in the 3D space at any time could be uniquely determined by palm normal vector $\vec{n}$ and palm direction vector $\vec{h}$. Let $\vec{m}=\vec{h}\times \vec{n}$, then a new coordinate system $\left[{\vec{h}}_{t},{\vec{n}}_{t},{\vec{m}}_{t}\right]$ can be obtained to represent the palm posture in frame *t*. We take the initial data frame of the dynamic gesture as the fixed coordinate system and denote it as $\left[{\vec{h}}_{t},{\vec{n}}_{t},{\vec{m}}_{t}\right]$. So, the change in palm posture between the current frame and the first frame can be represented with three Euler angles:

(2) $$\begin{array}{c} \left\{\begin{array}{c} \psi_{t}=\mathrm{arc}\tan\left(-\frac{\left({\vec{n}}_{1}\cdot {\vec{h}}_{t}\right)}{\left({\vec{n}}_{1}\cdot {\vec{n}}_{t}\right)}\right),\\ \phi_{t}=\mathrm{arc}\sin\left({\vec{n}}_{1}\cdot {\vec{m}}_{t}\right),\\ \theta_{t}=\mathrm{arc}\tan\left(-\frac{\left({\vec{h}}_{1}\cdot {\vec{m}}_{t}\right)}{\left({\vec{m}}_{1}\cdot {\vec{m}}_{t}\right)}\right). \end{array}\right. \end{array}$$

#### 3.2.2. Bending Angle of Fingers

**Figure 2:**
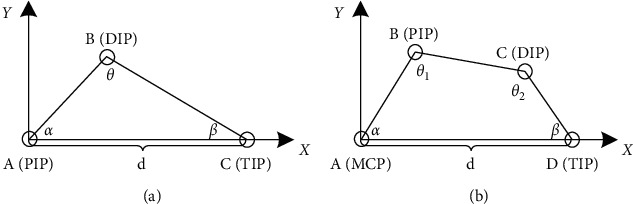
Simplified planar model of fingers: (a) triangular model for the thumb; (b) quadrilateral model for the other four fingers. MCP, PIP, DIP, and TIP represent metacarpophalangeal point, proximal interphalangeal point, distal interphalangeal point, and fingertip, respectively.

As we mainly focus on the bending angle of the finger, the thickness of the finger could be regarded as useless, and then, each finger can be simplified to a planar model, as shown in Figure 2. Based on the two models, Hong et al. [24] propose a method to estimate the hand's attitude or instead bending angle of fingers and coordinates of joint points. At all conditions, their method require a merely total length of the finger *l*_*i*_, visible length of the finger *d*_*i*_, and several constraint constants. Combining with their research, we define the finger bending angle as

(3) $$\begin{array}{c} \omega_{i}=\left\lfloor 100\times \left(\frac{d_{i}}{l_{i}}\right)\right\rfloor ,\;i=1,\ldots ,5, \end{array}$$

where *d*_*i*_ can be obtained directly from LM and *l*_*i*_ equals to *d*_*i*_ when the finger is straight.

In equation (3), *l*_*i*_ is used for normalization in order to make the approach robust to people with hands of different sizes. For *l*_*i*_, a simple method is proposed to calibrate before data acquisition. The user keeps his/her palm plane parallel to LM and open fingers as straight as possible. When data of total continuous frames satisfy (1) *n*_*y*_⟵0.94 and (2) $\left|{\vec{f}}_{i}\cdot \vec{n}\right|<0.008,i=1,\ldots ,5$, up to 30, the obtained visible lengths of five fingers could be recorded as total lengths, where *n*_*y*_ is the component of normal vector $\vec{n}$ along the *Y*-axis direction in the LM coordinate system.

#### 3.2.3. Opening Angle of Fingers

The other descriptor for the fingers is the opening angle between fingers. As mentioned above, every single finger can be modeled on a plane. Thus, the problem of computing the angle between two fingers can convert to one calculating the angle between two planes. Here, the plane consists of $\vec{h}$, and $\vec{n}$ is taken as the benchmark plane in the computation. Let $\vec{h}\times \vec{n}$ and ${\vec{f}}_{i}\times \vec{n}$ be the normal vector of the benchmark plane and finger planes, respectively. So, the opening angle can be calculated as follows:

(4) $$\begin{array}{c} \gamma_{i}=\text{arc }\cos\left(\frac{\left({\vec{f}}_{i}\times \vec{n}\right)\cdot \left(\vec{h}\times \vec{n}\right)}{\left\|\left({\vec{f}}_{i}\times \vec{n}\right)\right\|\times \left\|\left(\vec{h}\times \vec{n}\right)\right\|}\right),\;i=1,\ldots ,5. \end{array}$$

#### 3.2.4. Trajectory Feature

A specific and meaningful trajectory usually accompanies some dynamic gestures, such as circling with a finger (like G5). So, the paper considers the path of the dynamic gesture and extracts a simplified feature for gesture recognition. When LM works, it can detect the palm center's return space coordinates with high accuracy and stability. So the moving trajectory of a hand can be expressed by a series of discrete points. The paper projects the gesture trajectory onto the LM's principal gesture plane, i.e., the XOZ plane. The detailed feature extraction processes are as follows:

1. Let (*x*_1_, *z*_1_),…, (*x*_*T*_, *z*_*T*_) be the discrete points of the 2D gesture trajectory, then the central point *p*_*o*_(*x*_*o*_, *z*_*o*_) of these points can be expressed as follows:

(5) $$\begin{array}{c} p_{o}\left(x_{o},z_{o}\right)=\left(\frac{1}{T}\sum_{t=1}^{T}x_{t},\frac{1}{T}\sum_{t=1}^{T}z_{t}\right). \end{array}$$

(2) Any point *p*_*t*_(*x*_*t*_, *z*_*t*_) and *p*_*o*_(*x*_*o*_, *z*_*o*_) form a vector of $\vec{p_{o}p_{t}}$ together with the central point *p*_*o*_ as the starting point. Then, the norm of $\vec{p_{o}p_{t}}$ and the direction angles between $\vec{p_{o}p_{t}}$ and the *X*-axis can be represented as follows:

(6) $$\begin{array}{c} \left\{\begin{array}{c} d_{t}\left(p_{t},p_{o}\right)=\sqrt{{\left(x-x_{o}\right)}^{2}+{\left(z-z_{o}\right)}^{2}},\\ \varphi_{t}\left(p_{t},p_{o}\right)=\frac{180}{\pi }\times \tan^{-1}\frac{\left(z_{t}-z_{o}\right)}{\left(x_{t}-x_{o}\right)}. \end{array}\right. \end{array}$$

**Figure 3:**
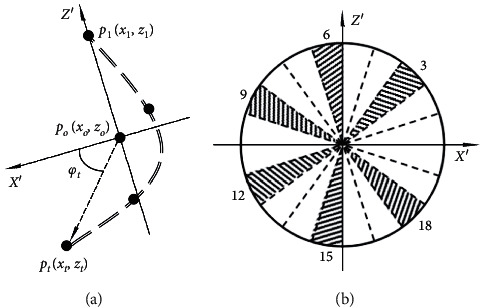
Schematic diagram of normalization process: (a) norm and direction angle of the vectors; (b) angular regions in the XOZ plane.

(3) Norm of the vectors *d*_*t*_ is normalized with the maximum norm *d*_max_, thus obtaining *δ*_*t*_. Besides, direction angles of the vectors *φ*_*t*_ are converted into codes *ψ*_*t*_ according to the angular regions, as shown in Figure 3. *δ*_*t*_ and *ψ*_*t*_ can be computed as follows:

(7) $$\begin{array}{c} \left\{\begin{array}{c} \delta_{t}=\left\lfloor 20\times \left(d_{t}\frac{\left(p_{t},p_{o}\right)}{d_{\max}}\right)\right\rfloor ,\\ \psi_{t}=\left\lfloor \varphi_{t}\frac{\left(p_{t},p_{o}\right)}{18^{{}^{\circ}}}\right\rfloor +1. \end{array}\right. \end{array}$$

Before coding the direction angle *φ*_*t*_, we change the coordinate system from the original LM one into the coordinate system, as shown in Figure 3(a), the z′ axis of which always points from the central point *p*_0_ to the first point *p*_1_. The obtained trajectory feature *δ*_*t*_ and *ψ*_*t*_ are of scale and rotation invariance based on the operation plane.

**Figure 4:**
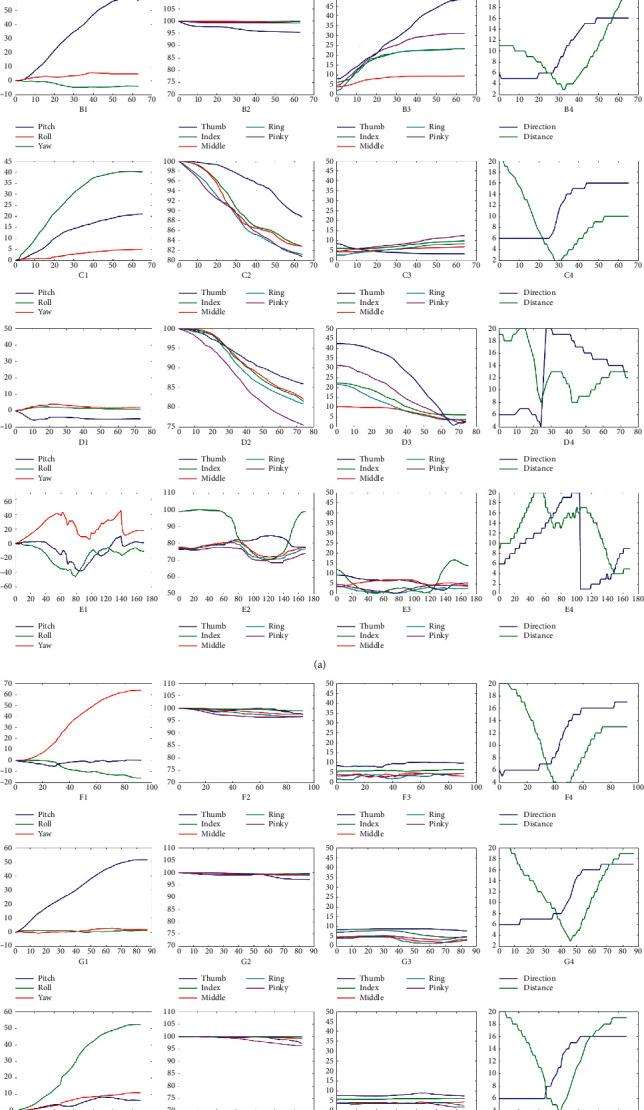
Features of the gestures in the LMC-Gesture3D training dataset.

Select typical data once for each gesture in the LM-Gesture3D dataset and build their feature diagrams, as shown in Figure 4. Each row in Figure 4 corresponds sequentially to one of the gestures in the LM-Gesture3D. Four descriptions in each row from left to right are palm posture, finger bending angle, finger opening angle, and trajectory, respectively. It is not hard to see that each feature diagram depicts how its corresponding gesture is performed nicely. Gesture with complicated changes usually corresponds to complex feature curves, and vice versa. Different gestures may have similar features. The palm posture feature of G1–G3, for example, is similar to that of G6–G8 finger bending angle, and finger opening angle of G6–G8 is similar to each other. Therefore, it is not easy to distinguish these gestures just with a single feature. Of course, there are some gestures with significantly different features like G1 and G2. So, there is no misrecognition between G1 and G2.

There may be some more distinguishing features that can improve the recognition rate as well as reduce the computation cost for a given gesture. However, considering eight kinds of gestures in LM-Gesture3D that have obvious similarities, we prefer to select a feature set with completeness and redundancy that meets the requirements of unified modeling and recognizes the gestures. According to the description of Figure 4, some features in the defined feature set are similar to each other for different gestures. However, there are some distinct features that are also included in the defined feature set. So, on the whole, the collected LM-Gesture3D or other more kinds of dynamic gestures can be adequately represented and distinguished by the defined four types of features.

**Figure 5:**
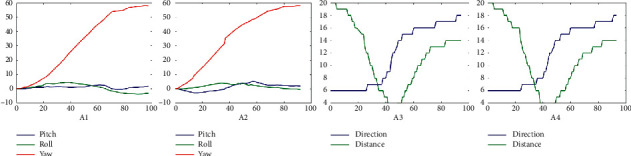
Palm posture and trajectory feature diagrams of gesture G6. a1 and a2 are the feature diagrams of palm posture corresponding to two gestures; a3 and a4 are the feature diagrams of trajectory corresponding to two gestures.

In all four features, finger bending angle and finger opening angle are not affected by acquisition direction. To verify whether the rest two kinds of features are rotation invariance, we obtain the hand data of the gesture G6 from an experimenter, who is asked to make the gesture G6 twice during the collecting period. Then, we extract the posture feature and trajectory feature from the collected hand data and draw the feature curves, as shown in Figure 5.

### 3.3. Feature Sequence Clustering

As shown in Table 2, in a single data frame of a gesture, four features can be represented by *m*_*i*_(*i*=1,2,3,4) dimensional vectors, respectively. Accordingly, each feature in *T* data frames of a dynamic gesture forms *T* × *m*_*i*_ dimensional vector sequences. In order to build the model of discrete HMM, *K*-means algorithm [25] is used to cluster the feature vector in the sequence. After clustering a feature vector into *q* class, the feature vector sequence can be expressed as *O*={*o*_1_,…, *o*_*t*_,…, *o*_*T*_}, where *o*_*t*_=1,…, *q* indicates that the feature vector is closest to the cluster center numbered *o*_*t*_. In the paper, the cluster number *q* of four kinds of features is shown in Table 2.

In short, we take the discrete feature sequence composed of cluster tags as inputs of the discrete HMM. Therefore, both the sample data for HMM training stage and the gesture data for HMM recognizing need to go through the steps of feature extraction and clustering.

## 4. Gesture Modeling and Recognition

### 4.1. Recognizing Flow

**Figure 6:**
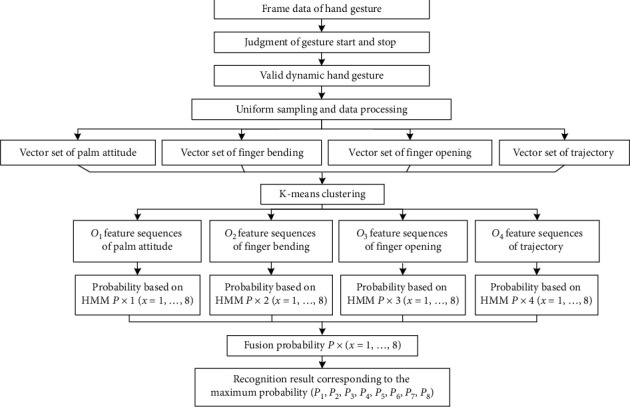
Implementation flow of the proposed method for recognizing dynamic gesture.

The recognizing process of gesture is shown in Figure 6, which can be divided into two parts. The first part deals with the accurate gesture segmentation and four features extraction and quantification. The second part includes HMM model training and gesture recognition, both of which are based on the premise of feature sequences extraction.

The formal features of HMM can be expressed with a 5-tuple (Ω_*X*_, Ω_*O*_, **A**, **B**, *π*), where Ω_*X*_={*q*_1_,…, *q*_*N*_} is a finite set Markov chain state, and *N* is the number of states; Ω_*O*_={*V*_1_,…, *V*_*M*_} is a finite set of observation symbols, and *M* is the number of symbols. **A**=(*a*_*ij*_)_*N*×*N*_ is the matrix of state transition probability, **B**=(*b*_*ij*_)_*N*×*M*_ is the matrix of observation probability, and *π*=(*π*_1_,…, *π*_*N*_) is the initial state probability distribution.

### 4.2. HMM Training

Unlike common one HMM for one kind of gesture modeling pattern, we build one HMM model for each feature, which means that 4 HMM models are adopted to achieve the recognition of each performed unknown gesture. Taking LM-Gesture3D for example, the designed 8 gestures are denoted by *g*_*u*_, *u*=1,…, 8; then, for the feature sequence *S*_*u*_^*v*^(*v*=1,…, 4 ) of gesture *g*_*x*_, the following HMM modeling processes are carried out:

1. HMM initialization: according to Table 1, in the paper, *N* is set to be 6. The number of observation symbols *M* is set as the same value of the number of cluster centers shown in Table 2; the initialization model parameters are described as *λ*_*u*_^*v*^=(**A**, **B**, *π*).
2. HMM parameters revaluation: assume that the feature sequence *S*_*u*_^*v*^ consists of *K* observation sequences *O*^(*k*)^, where *k*=1,…, *K*, and each observation sequence could be represented as *O*^(*k*)^={*O*_1_^(*k*)^,…, *O*_*T*_^(*k*)^}.

For computing ${\bar{\pi }}_{i}$, ${\bar{a}}_{ij}$, and ${\bar{b}}_{js}$, respectively, the observation sequence *O*^(*k*)^ and the original model parameter *λ*_*u*_^*v*^ are substituted into the reestimation equations as follows:

(8) $$\begin{array}{c} {\bar{\pi }}_{i}=\sum_{k=1}^{K}\frac{a_{1}^{k}\left(i\right)\beta_{1}^{k}\left(i\right)}{P\left(\left.O^{\left(k\right)}\right|\lambda \right)},\\ {\bar{a}}_{ij}=\frac{\sum_{k=1}^{K}\sum_{t=1}^{T_{k}-1}a_{t}^{k}\left(i\right)a_{ij}b_{j}\left(O_{i+1}^{\left(k\right)}\right)b_{t+1}^{k}\left(j\right)/P\left(\left.O^{\left(k\right)}\right|\lambda \right)}{\sum_{k=1}^{K}\sum_{t=1}^{T_{k}-1}a_{t}^{k}\left(i\right)\beta_{t+1}^{k}\left(i\right)/P\left(\left.O^{\left(k\right)}\right|\lambda \right)},\\ {\bar{b}}_{js}=\frac{\sum_{k=1}^{K}\sum_{\begin{array}{c} t=1\\ o_{t}^{k}=v_{s} \end{array}}^{T_{k}-1}a_{t}^{k}\left(j\right)\beta_{t}^{k}\left(j\right)/P\left(\left.O^{\left(k\right)}\right|\lambda \right)}{\sum_{k=1}^{K}\sum_{t=1}^{T_{k}-1}a_{t}^{k}\left(j\right)\beta_{t}^{k}\left(j\right)v/P\left(\left.O^{\left(k\right)}\right|\lambda \right)}, \end{array}$$

where 1 ≤ *i*, *j* ≤ *N*.

Thus, a new model ${\bar{\lambda }}_{u}^{v}=\left(\bar{\pi },\bar{A},\bar{B}\right)$ is obtained. The above process would be repeated until the parameters in two adjacent iterations meet as follows:

(9) $$\begin{array}{c} \left|\log P\left(\left.O\right|{\bar{\lambda }}_{u}^{v}\right)-\log P\left(\left.O\right|\lambda_{u}^{v}\right)\right|<\varepsilon , \end{array}$$

where *P*(*O*|*λ*) is calculated from the forward-backward algorithm, which indicates the occurrence probability of the observation sequence *O* under the parameter *λ*, and *ε* is the predefined convergence threshold.

The final model parameter ${\bar{\lambda }}_{u}^{v}$ is the optimal parameter of feature sequence *S*_*u*_^*v*^, that is, the single feature HMM of its corresponding gesture. By repeating the above modeling process for each feature sequence *S*_*u*_^*v*^ of 8 dynamic gestures, we can obtain 32 single-feature HMM models in total.

### 4.3. Gesture Recognition with HMM Fusion

In the stage of gesture recognition, once original data of an unknown and valid dynamic gesture are obtained, it would be first extracted into 4 observation sequences *O*_1_, *O*_2_, *O*_3_,  and *O*_4_. Then, the forward-backward algorithm is used to calculate the occurrence probability $P\left(\left.O_{1}\right|{\bar{\lambda }}_{u}^{1}\right)\left(u=1,\ldots ,8\right)$ of the observation sequence under 8 single feature HMM ${\bar{\lambda }}_{u}^{1}\left(u=1,\ldots ,8\right)$. Similarly, the occurrence probability of the observed sequence *O*_2_, *O*_3_,  and *O*_4_ under their corresponding HMM ${\bar{\lambda }}_{u}^{2},{\bar{\lambda }}_{u}^{3},\text{ and }{\bar{\lambda }}_{u}^{4}$ can be obtained. For demonstration purposes, we represent the occurrence probabilities $P\left(\left.O_{v}\right|{\bar{\lambda }}_{u}^{v}\right)$ as *P*_*uv*_(*v*=1,2,3,4).

We present an algorithm of weighted probability fusion to compute the probability that an unknown gesture belongs to the gesture *u* in LM-Gesture3D as follows:

(10) $$\begin{array}{c} P_{u}^{\text{F}}=\sum_{v=1}^{4}\omega_{uv}P_{uv}, \end{array}$$

where *ω*_*uv*_(0 ≤ *ω*_*uv*_ ≤ 1 and ∑_*v*=1_^4^*ω*_*uv*_=1) is the weight of feature *v* corresponding to the gesture *u*.

According to equation (10), there are 8 calculation results, in which the maximum is regarded as the recognition result of the unknown gesture.

The paper employs least square method (LSM) to determine *ω*_*uv*_ in equation (10). Here is a brief introduction to the LSM weight method. Firstly, we calculate the probabilities of four features for all samples in the training dataset and can obtain **P**^*m*^={**P**_1_^*m*^, **P**_2_^*m*^,…, **P**_8_^*m*^}(*m*=1,…, *L*), where *L* is the number of samples and **P**_*u*_^*m*^=(*P*_*u*1_^*m*^, *P*_*u*2_^*m*^, *P*_*u*3_^*m*^, *P*_*u*4_^*m*^). Secondly, for the gesture *u* in LM-Gesture3D, if the sample *m* belongs to it, we set the probability of the sample *m* corresponding to the gesture *u* as follows:

(11) $$\begin{array}{c} P_{{}_{m,u}}^{\text{F}}=\sum_{v=1}^{4}\omega_{uv},\\ P_{uv}^{m}=\omega_{u},\\ {\left(P_{u}^{m}\right)}^{\text{T}}=p_{s}. \end{array}$$

Else, the probability of the sample *m* corresponding to the gesture *u* is set to be as follows:

(12) $$\begin{array}{c} P_{{}_{m,u}}^{\text{F}}=\sum_{v=1}^{4}\omega_{uv}P_{uv}^{m}=\omega_{u}{\left(P_{u}^{m}\right)}^{\text{T}}=\frac{\left(1-p_{s}\right)}{7}, \end{array}$$

where *p*_*s*_(0.5 ≤ *p*_*s*_ ≤ 1) is a set probability.

Calculating the probabilities of all samples corresponding to the gesture *u*, we can obtain the following formula:

(13) $$\begin{array}{c} \omega_{u}\left[\begin{array}{c} {\left(P_{u}^{1}\right)}^{\text{T}}\\ {\left(P_{u}^{2}\right)}^{\text{T}}\\ \vdots \\ {\left(P_{u}^{V}\right)}^{\text{T}} \end{array}\right]=\left[\begin{array}{c} P_{u,1}^{\text{F}}\\ P_{u,2}^{\text{F}}\\ \vdots \\ P_{u,V}^{\text{F}} \end{array}\right]. \end{array}$$

We use the least square method (LSM) to compute *ω*_*u*_=(*ω*_*u*1_, *ω*_*u*2_, *ω*_*u*3_, *ω*_*u*4_) in equation (15). Finally, *ω*_*u*_ is normalized to *ω*_*u*_′=(*ω*_*u*1_′, *ω*_*u*2_′, *ω*_*u*3_′, *ω*_*u*4_′). *ω*_*u*_′ is the weight vector of the fusion model.

## 5. Experiments

To test the performance of the proposed method, several experiments are carried out on a desktop PC with an Intel Core i5-3230M processor and 4 Gb of RAM, and the software environment consists of Visual Studio 2013, Leap Motion SDK 2.3.1 + 3154, and MATLAB 2012a.

### 5.1. LM-Gesture3D Recognition Experiment

We select four participants with certain experiences in robot teleoperation to join the experiment. Each participant is asked to imitate each gesture in LM-Gesture3D 40 times repeatedly, and LM samples their gestures. So, there are 160 samples of each gesture.

To verify the feasibility of the proposed method, we define the recognition rate as follows:

(14) $$\begin{array}{c} \text{RR}=\frac{N_{\text{Rec}}}{M_{\text{Sam}}}\times 100\%, \end{array}$$

where *N*_Rec_ is the number of gestures correctly recognized and *M*_Sam_ is the total number of gestures recognized.

**Figure 7:**
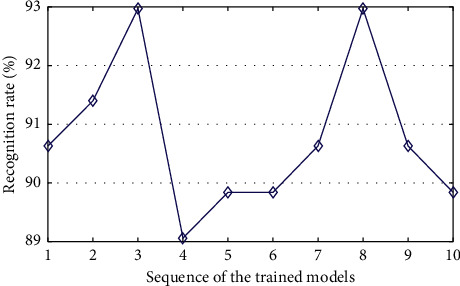
Recognition accuracies of LM-Gesture3D by using *k*-fold cross-validation.

Firstly, we use *K*-fold cross-validation to evaluate the recognition performance and stability of the proposed method. In this experiment, *K* is set to be 10. So, each subset has 128 samples.  Figure 7 is the result of *K*-fold cross-validation, which shows that the recognition rates of different trained models range from 89.8% to 92.9%. The fluctuation ranges of recognition rates of all 10 trained HMM models are about 3%, which shows that the proposed method has a good generalization ability. The average recognition rate of all 10 trained HMM models is about 90.8%, which indicates that the proposed method has a good recognition performance.

Furthermore, we analyze the recognition performance of the proposed method for different types of gestures in LM-Gesture3D. We randomly select 60 samples of each gesture as the testing set and the remaining samples as the training set.  Table 3 shows the recognizing results. From the table, we can see that our method has a good representation of the 8 dynamic gestures with the average recognition rate of about 90.6%. The recognition rates for all gestures fluctuate slightly between 88.3% and 91.7%. The recognition rates of G4 and G6–G8 are higher than those of G1–G3 and G5. The reason is that these gestures are relatively simpler and easier for different users to repeat, while the participant's individual habits easily influence G1–G3 and G5. In addition, gestures G1–G3 are easily confused with G6–G8, respectively.

In general, the recognition results are jointly determined by four kinds of features, and our method based on multiple features and HMM can represent most kinds of complex gestures, which proves that our method is effective.

### 5.2. Dynamic Gesture Recognition Experiments

**Figure 8:**
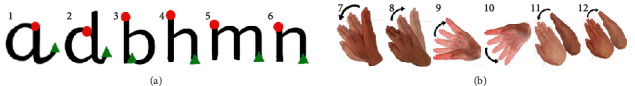
Gestures in letter-gesture set and waving-gesture set: (a) 6 kinds of gestures in letter-gesture set; (b) 6 kinds of gestures in waving-gesture set.

This experiment mainly tests our method's recognition rate for two kinds of relatively simple dynamic gestures, which are named letter-gesture dataset and the waving-gesture dataset, respectively. As shown in Figure 8(a), letter-gesture set consists of 6 gestures numbered 1 to 6, which are similar to each other. The waving-gesture dataset contains the rest 6 gestures shown in Figure 8(b). It can be seen that the main feature of two gesture sets is trajectory feature and palm posture feature, respectively.

The gestures in the experiment are sampled from four participants. Each participant is asked to repeat each gesture 50 times. When collecting the letter-gesture dataset, each participant keeps the shape as unchanged as possible and parallel to the horizontal plane of LM. Each gesture's obtained data are further divided into 120 sets of training samples and 80 sets of testing samples.

Chen et al. [17] propose a rapid early recognition system based on SVM to achieve multiclassification among the 36 dynamic gestures (the 3D motion trajectory of the numbers and the alphabet). Chen's method uses LM to capture 3D motion trajectories of the gestures, which is the same as our method. In Chen's method, the orientation angle is utilized as a unique feature of the gesture trajectory projected into the XOZ plane. It is quantized by dividing it by 45° and coded from 1 to 9, which is similar to our method. Chen's method is also used to recognize the gestures in the letter-gesture dataset.

**Figure 9:**
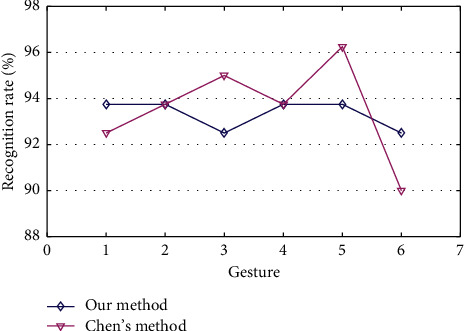
Results of using our method and Chen's method to recognize gestures from letter-gesture dataset.

Figure 9 shows the recognition results of our method and Chen's method. Our method and Chen's method get the average recognition rates of 96.0% and 93.5%, respectively. Two approaches have very similar recognition rates. However, the fluctuation of our method's recognition rate with LSM weights is smaller than that of Chen's approach. It shows that our method has better recognition stability than Chen's method.

In addition, the directional code extracted by Chen's method is determined by two neighboring points on the trajectory. In contrast, that of our method is determined by the trajectory points and the central point. At the same time, we also introduce a distance feature. Therefore, the extracted trajectory feature by our method is not affected by the amplitude of the gesture and is of rotation invariance.

Based on the above analysis, we believe that our method performs better than Chen's method.

The waving direction of gestures 7–10 in the waving-gesture dataset is from upper right to lower left, from upper left to lower right, from top to bottom, and from bottom to top, respectively. And other gestures 11 and 12 make roughly 90° clockwise and counterclockwise rotations, respectively. It can be seen that this kind of dynamic gesture could be distinguished easily once using palm posture features. We carry an experiment to test the recognition performance of our method aiming at the 6 kinds of gestures. In the experiment, the method of data acquisition and processing is the same as that in the experiment of the letter-gesture dataset.

Pan et al. [26] present a combination method based on rule-based classification and SVM recognizes the gestures, which also use LM to capture real-time frame data of hand motion and define a 14-dimensional feature set including the absolute pose of hand in the 3D coordinate system and the pose changes in the hand between the two frames. Pan's method is also used to recognize the gestures in the waving-gesture dataset.

**Figure 10:**
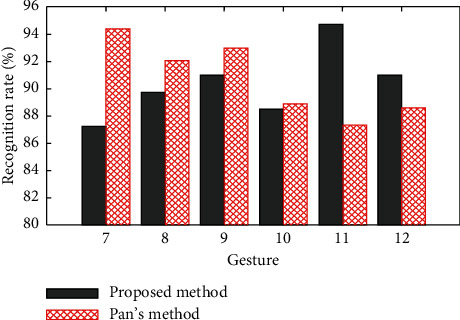
Results of using our method and Pan's method to recognize the waving-gesture dataset.

Figure 10 shows the recognition results of our method and Pan's method. The recognition rates of two methods for gestures 7–12 are all over 90, and the average recognition rates are 90.4% and 90.8%, respectively. The average recognition rate of Pan's method is slightly higher than that of our method.

Compared with our method, Pan's method will lead to more computational costs because it selects high dimension features and adopts a two-step recognizing strategy. Our method has not only a high recognition rate but also has the rotational invariance for selecting the rotation angle based on the initial posture of hand as features. Our method has a good effect on recognizing the wave or rotation gestures, such as those in the waving-gesture dataset.

In addition, all three methods above use LM to sample the gestures. The data of the features defined by three methods can be obtained quickly and accurately by LM. But adopting the camera approach, we have to depend on the hand area feature to recognize the gestures, which is more complex and challenging. Hence, we can conclude that LM brings excellent benefits to our research.

### 5.3. Generalization Experiment

**Figure 11:**
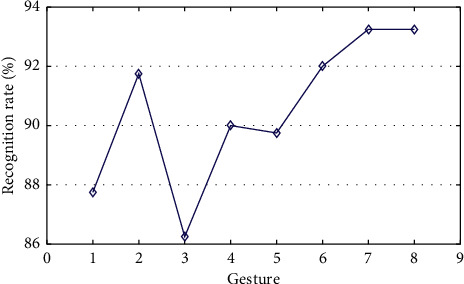
Recognition rate on nontrainer's gestures in LM-Gesture3D.

A generalization experiment is carried out to verify the adaptability of our method to nonstandard gestures. We select four inexperienced participants for the experiment. In the experiment, each participant is asked to repeat each gesture from LM-Gesture3D 40 times. A total of 1280 different gestures are sampled, which are recognized by the built HMM mode and the same weights in the LM-Gesture3D recognition experiment. The average recognition rate of 90.5% shown in Figure 11 is very similar to that of the LM-Gesture3D recognition experiment. So, the method is adaptable to different nonstandard gestures and has a good generalization ability.

We defined positive prediction value (PPV) and accuracy (ACC) of the gesture Gi (*i* = 1, 2,…, 8) as follows:

(15) $$\begin{array}{c} \text{PPV}=\frac{{\text{T}}_{\text{G}i}}{{\text{T}}_{\text{G}i}{+\text{F}}_{\text{G}i}}, \end{array}$$

where T_G*i*_ is the number of gesture Gi correctly recognized and F_G*i*_ is the number of other seven gestures that are incorrectly recognized as Gi.

(16) $$\begin{array}{c} \text{ACC}=\frac{{\text{T}}_{\text{G}i}}{{\text{T}}_{\text{G}i}+\sum_{1\leq j\leq 8,j\neq i}{\text{F}}_{\text{G}j}}, \end{array}$$

where F_G*i*_ is the number of gesture Gi that is incorrectly recognized as gesture Gj.

Table 4 shows the confusion matrix of the generalization experiment using the proposed method. According to Table 4, except for G5 with a PPV of about 0.96, the recognition precisions for the other seven gestures have a small difference ranging from 0.89 to 0.91.

### 5.4. Comparison Experiment with Other HMM-Based Methods

Here, we compare the recognition performance of the proposed method with other recognition methods based on HMM.

The authors in [11] define three features, including handshape, palm trajectory, and distance from the camera to extract the hand model from image features. It proposes a combinatorial method based on HMM and BPNN. The HMM-BPNN method uses the classical HMM to evaluate and decide the dynamic gesture features and, then, uses the BP neural network to classify the input state sequence.

In this experiment, the experimental samples are from the LM-Gesture3D recognition experiment in Section 5.1, from which 60 samples of each gesture and the remaining samples are randomly selected as the testing and training sets, respectively. The experiment is divided into two parts, including the feature testing and algorithm testing.

The feature testing experiment uses the features defined in the paper [12] to describe the gestures and analyze the HMM-BPNN method's recognition rate.  Table 5 shows the recognition results of the experiment. From the table, we can see that the HMM-BPNN method has an average recognition rate of only about 50.83% for 8 dynamic gestures. Moreover, for different types of gestures, its recognition rate fluctuates greatly. The main reason for the low recognition rate of the HMM-BPNN method for the gestures in LM-Gesture3D is that the three types of 2D features defined by the method are only suitable for representing simple and highly differentiated gestures but cannot fully represent complex and highly similar gestures, such as G5.

The algorithm testing experiment uses the features defined by our method to describe the gestures and analyze the recognition rate of the HMM-BPNN method again.  Table 6 shows the recognizing results of the experiment.

From Table 6, we can see that the HMM-BPNN method has an average recognition rate of about 80.83% for 8 dynamic gestures. The recognition rate of the experiment is 30% higher than that of the feature testing experiment. Moreover, for different types of gestures, its recognition rate fluctuates less. The results show that the paper's features can more effectively represent complex gestures in LM-Gesture3D than that of the HMM-BPNN method.

For the same gesture samples and the same defined features, the recognition rate of our method, shown in Table 3, is more than 90%, which is about 10% higher than that of the HMM-BPNN method. We think there are two main reasons for the relatively low recognition rate of the HMM-BPNN method. Firstly, the input of the BPNN classifier is decided by a maximum assessment of the probabilities of the trained HMM models of four types of features, which does not consider the interference between similar features. Secondly, the BP neural network is prone to fall into local minima, which increases the risk of misrecognition when different sample features have significant similarities.

## 6. Conclusion

In the paper, a fusion recognition method based on multiple features and HMM for the dynamic gesture is proposed. We consider both the change in handshape and moving trajectory and build four sorts of hand features with the advantages of being straightforward, simple, and rotation invariance, which bring better operation naturalness and flexibility for the operators. What is more, it offers a further expansion of more kinds of complex dynamic gestures by using these features. For each feature, we have built its corresponding HMM. In the recognition stage, we innovatively present a weighted fusion algorithm to calculate the occurrence probabilities and get the final recognition result. In the above way, the result is not easily affected by a particular feature.

The experimental results show that the proposed method is suitable for relatively simple dynamic gestures like letter gestures and waving gestures. Still, it also has strong robustness for complex dynamic gestures like LM-Gesture3D. The average recognition rate of the proposed method for LM-Gesture3D is up to 90.6%. Besides, the average recognition rate for inexperienced participants is about 90%. These results demonstrate the usability and feasibility of the proposed method.

Like other gesture recognition methods, the proposed method inevitably has certain limitations, and a more in-depth study needs to be carried out. Firstly, as we have adopted four HMMs for each gesture recognition, the algorithm's efficiency remains to be raised. Secondly, we have not yet done more research on the adaptive weight method and their further impact on the recognition rate, which will also be a future research direction.

## Figures and Tables

**Table 1:** Definition of LM-Gesture3D dataset.

Num	Gestures	Num	Gestures
G1	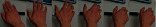	G2	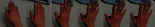
G3	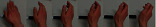	G4	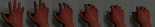
G5	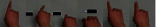	G6	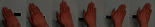
G7	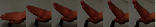	G8	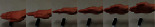

**Table 2:** Number of cluster centers of four features.

Feature	Feature vector	*q*
Palm posture	[*ψ*_*t*_, *φ*_*t*_, *θ*_*t*_]	16
Finger bending angle	[*ω*_1_, *ω*_2_, *ω*_3_, *ω*_4_, *ω*_5_]	14
Finger opening angle	[*γ*_1_, *γ*_2_, *γ*_3_, *γ*_4_, *γ*_5_]	10
Trajectory	[*δ*_*t*_, *ψ*_*t*_]	10

**Table 3:** Recognition rate of the proposed method for the gestures in LMC-Gesture3D.

Gesture	G1	G2	G3	G4	G5	G6	G7	G8	RR
G1	54			3		3			90.0
G2		54					6		90.0
G3			53			3		4	88.3
G4	2		3	55					91.7
G5	3		3		55				90.0
G6	5					55			91.7
G7		6					54		90.0
G8			3				2	55	91.7

**Table 4:** Confusion matrix of the recognition results.

		Gesture samples								PPV
		G1	G2	G3	G4	G5	G6	G7	G8	
Recognition results	G1	140	1	3	5	2	2	1	1	0.90
	G2	2	146	3	2	3	2	3	2	0.90
	G3	1	2	138	3	3	2	1	1	0.91
	G4	6	2	3	144	2	1	1	1	0.90
	G5	1	1	1	1	143	1	1	0	0.96
	G6	7	1	2	2	2	147	2	2	0.90
	G7	1	5	3	1	3	3	150	3	0.89
	G8	2	2	7	2	2	2	1	150	0.89
ACC		0.88	0.91	0.86	0.90	0.89	0.92	0.94	0.94	0.90

**Table 5:** Recognition rate of the HMM-BPNN method of the feature test.

Gesture	G1	G2	G3	G4	G5	G6	G7	G8	RR
G1	35	7	3	5	1	4	2	3	58.3
G2	6	34	4	4	2	3	4	3	56.7
G3	5	5	30	5	2	4	4	5	50.0
G4	4	5	6	31	1	4	4	5	51.7
G5	5	4	3	5	25	5	5	6	41.7
G6	5	4	5	4	4	30	4	4	50.0
G7	4	6	4	4	2	4	29	7	48.3
G8	5	5	3	4	2	5	6	30	50.0

**Table 6:** Recognition rate of the HMM-BPNN-based method of the algorithm testing.

Gesture	G1	G2	G3	G4	G5	G6	G7	G8	RR
G1	49	2	3	2		3	1	1	81.7
G2	3	48	1	1		2	3	2	80.0
G3	2	1	50	1		2	1	2	83.7
G4	2	2	3	48		1	2	2	80.0
G5	3	2	2	1	48	2		2	80.0
G6	4	2	1	1		50	2	1	83.3
G7	1	4	1	1	1	2	48	2	80.0
G8	2	2	3	2	1		3	47	78.3

## Data Availability

The research library related to the dissertation will be established in GitHub (https://github.com/glchenwhut), where you can access the folders and find experimental data and lists.
